# Supplementary figures and images for: Environmental fungi from cool and warm neighborhoods in the heat island of Baltimore City show differences in thermal susceptibility and pigmentation
Source: ISME Commun. 2025 Oct 4;5(1):ycaf177. doi: 10.1093/ismeco/ycaf177 (PMC12551456; doi:10.1093/ismeco/ycaf177)

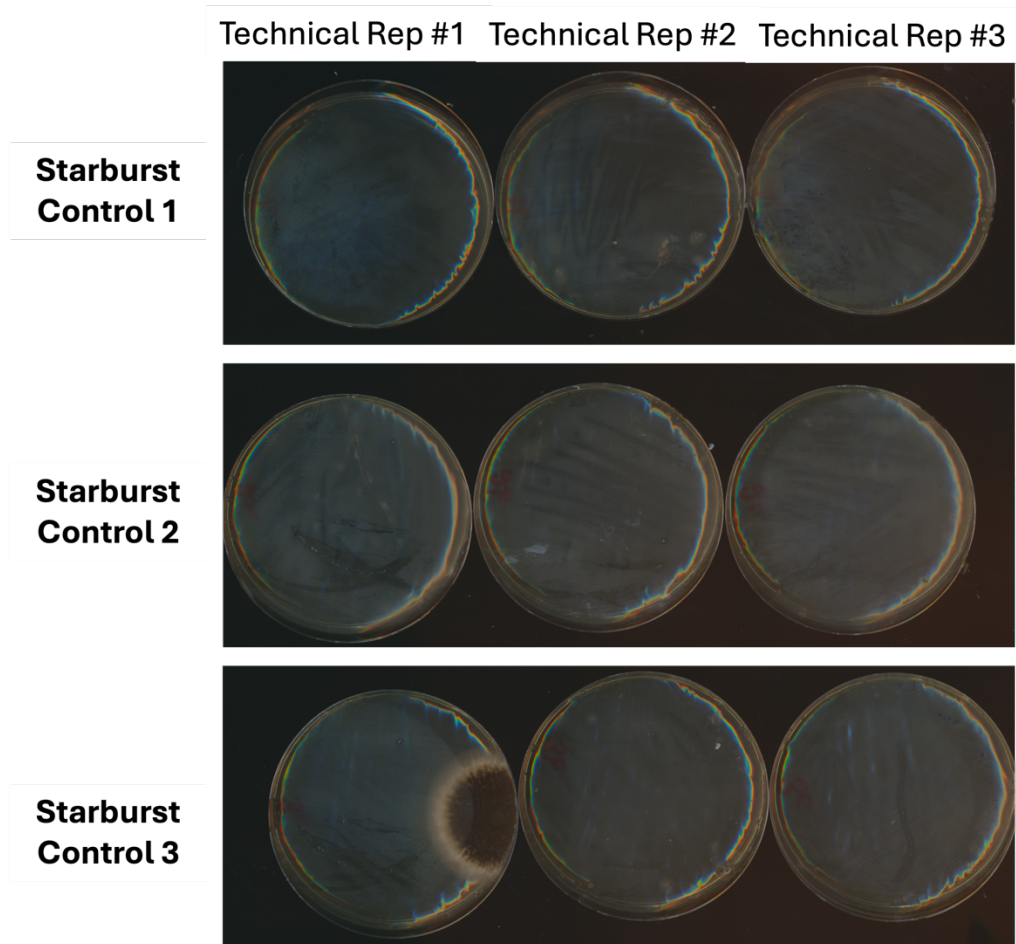

***Supplementary Figure 1. Agar Plates with Non-sidewalk control Starburst Growth.***

Supplement: Heat_Supplementary_Figure_1_ycaf177 [file heat_supplementary_figure_1_ycaf177.pdf]
