# Supplementary material for: Environmental fungi from cool and warm neighborhoods in the heat island of Baltimore City show differences in thermal susceptibility and pigmentation
Source: ISME Commun. 2025 Oct 4;5(1):ycaf177. doi: 10.1093/ismeco/ycaf177 (PMC12551456; doi:10.1093/ismeco/ycaf177)
Supplement: Heat_Supplementary_Figure_2_ycaf177 [file heat_supplementary_figure_2_ycaf177.pdf]

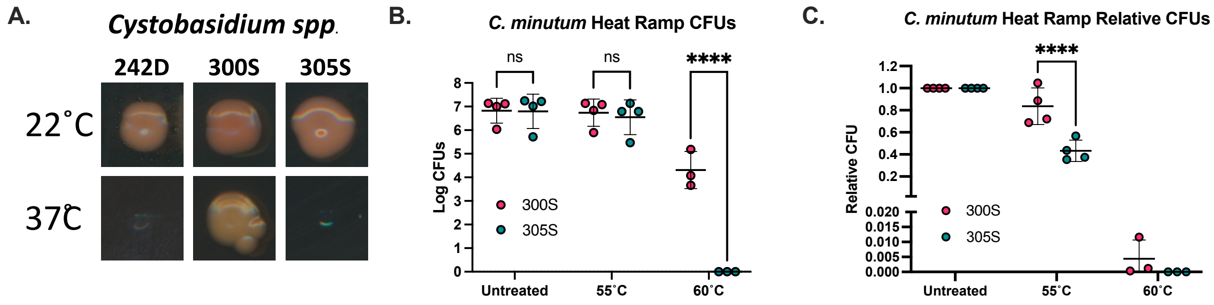

Supplementary Figure 2*. Thermotolerance of *Cystobasidium* spp. (A)** Cystobasidium spp. isolated from Site 2 dirt (242D) and Site 3 sidewalks (*305*S) were unable to grow at 37˚C, but *one* C. minutum isolate from Site 3 sidewalk (30*0*S) *was* thermotolerant at 37˚C. *C. minutum* from the sidewalk of Site 3 (300S) grows better than the other C. minutum isolated from the Site 3 sidewalk (305S) when exposed to gradual 55˚C heat ramp, while only 300S can grow following exposure to 60˚C heat ramp (Panels B and C). *S*ignificance of Two-Way ANOVA with multiple comparisons to the 300S for B and C at each condition with three biological replicates. Each dot represent CFUs from an individual biological replicate. * represents $p<0.0001$, ** represents $p<0.01$, and ns represents $p >0.05$.
