# Supplementary material for: Environmental fungi from cool and warm neighborhoods in the heat island of Baltimore City show differences in thermal susceptibility and pigmentation
Source: ISME Commun. 2025 Oct 4;5(1):ycaf177. doi: 10.1093/ismeco/ycaf177 (PMC12551456; doi:10.1093/ismeco/ycaf177)
Supplement: Heat_Supplementary_Figure_3_ycaf177 [file heat_supplementary_figure_3_ycaf177.pdf]

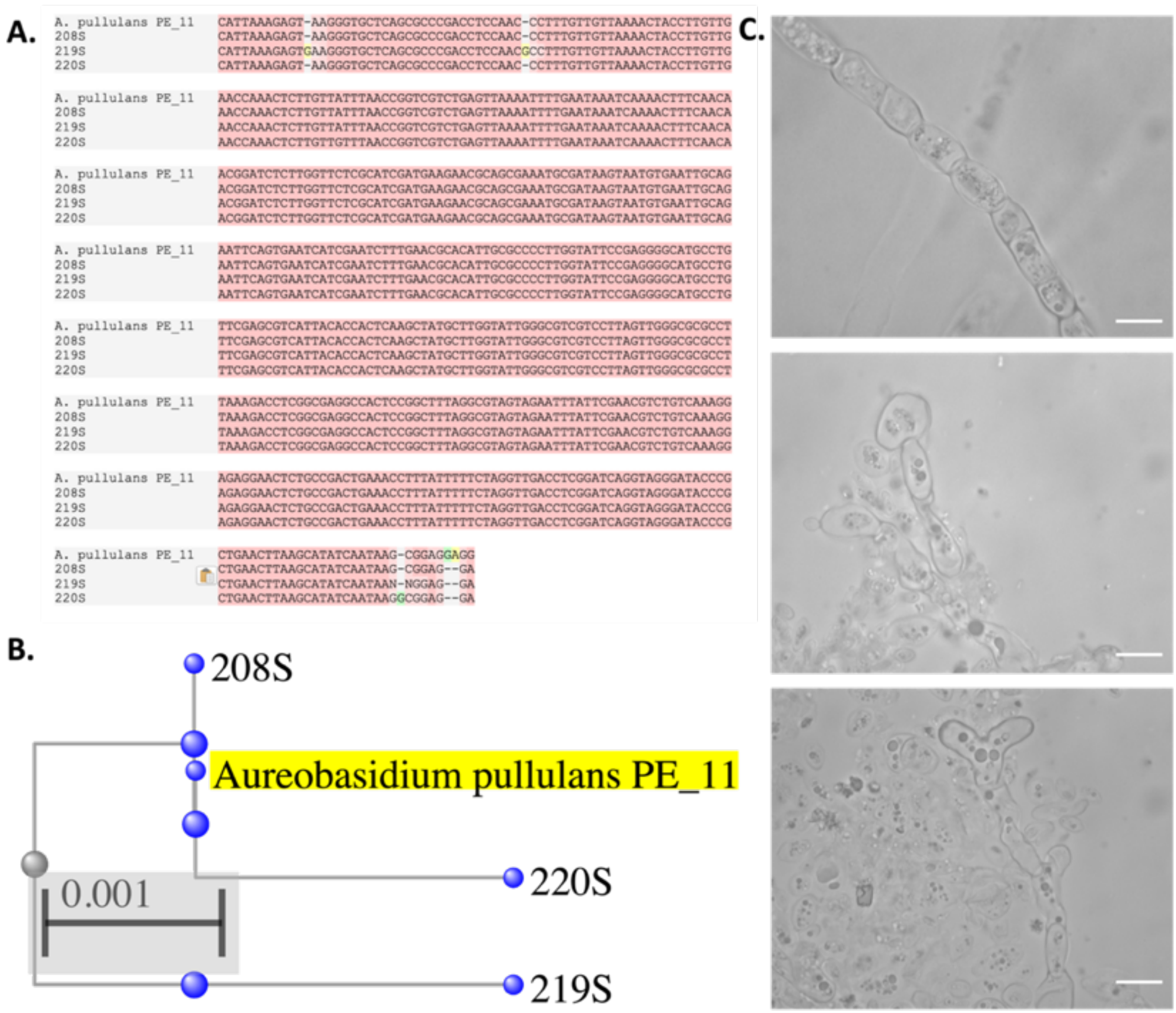

Supplementary Figure 3. Identification of *Aureobasidium pullulans* from High-Temperature Environment. Alignment of three of the 15 A. pullulans cultures isolated from Site 2 sidewalk (208S, 219S, 220S) to the A. pullulans PE_11 isolate (A), and their phylogenetic relationship (B). Microscopic images of the *A. pullulans* cultures show hyphal, yeast-like, and irregular shaped (C). Scale bar represents 20 μm.
